# The vaginal and uterine blood flow changes during the ovsynch program and its impact on the pregnancy rates in Holstein dairy cows

**DOI:** 10.1186/s12917-022-03444-9

**Published:** 2022-09-17

**Authors:** Heba A. Sharawy, AbdelRaouf O. Hegab, Engy F. Risha, Mohamed El-Adl, Walid T. Soliman, Mohamed A. Gohar, Reham A. Fahmy, Virginia M. Farag, Kazuhiko Imakawa, Fuller W. Bazer, Daniela James, Adel Zaghloul, Abdelnasser A. Abdalla, Mariam M. Rabie, Mohammed A. Elmetwally

**Affiliations:** 1 Department of Theriogenology, Center for Reproductive Biotechnology, Faculty of Veterinary Medicine, Mansoura University, Mansoura, 35516 Egypt; 2 Department of Clinical Pathology, Faculty of Veterinary Medicine, Mansoura University, Mansoura, 35516 Egypt; 3 Department of Biochemistry and Chemistry of Nutrition, Faculty of Veterinary Medicine, Mansoura University, Mansoura, 35516 Egypt; 4 Veterinary Department, Egyptian Armed Forces, Nasr City, Cairo, Egypt; 5 Oncology center, Faculty of Medicine, Mansoura University, Mansoura, 35516 Egypt; 6 Laboratory of Molecular Reproduction, Research Institute of Agriculture, Tokai University, Kumamoto, 862-8652 Japan; 7 Department of Animal Science, Texas A&M University, College Station, Texas USA; 8 Faculty of Agricultural Sciences, University of Applied and Environmental Sciences U.D.C.A., Bogota, Colombia; 9 Department of Surgery, Anesthesiology, and Radiology, Faculty of Veterinary Medicine, Mansoura University, Mansoura, 35516 Egypt; 10 Department of Internal Medicine, Infectious and Fish Diseases, Mansoura Veterinary Teaching Hospital, Mansoura University, Mansoura, 35516 Egypt

**Keywords:** Ovsynch, Vaginal blood flow, Uterine blood flow, pregnancy rates; Cows

## Abstract

**Aim:**

OvSynch is a hormonal protocol for synchronization of estrus and use of artificial insemination (AI) at an optimal time without adverse effects on the ovaries or uterus. This study investigated the use of noninvasive color Doppler ultrasound to assess changes in uterine and vaginal blood flow during the Ovsynch program for synchronization of estrus and its relation to the pregnancy rates in Holstein cows.

**Materials and methods:**

The experimental cows received an intramuscular dose of 10 μg of a GnRH analogue (G1), followed 7 days later with an intramuscular injection of synthetic prostaglandin F2α (P: PGF2α) analogue (500 μg cloprostenol sodium), and given a 10 μg, injection of the GnRH analogue (G2) i.m. 48 h after the PGF2α treatment, and the cows were bred 14-16 h after. Uterine and vaginal perfusion were investigated by performing transrectal Doppler ultrasonography of both the uterine and vaginal arteries in Holstein cows at different time points during the Ovsynch program to determine: peak systolic velocity (PSV), time-averaged maximum velocity (TAMV), the volume of blood flow (BFV), pulsatility index (PI), resistance index (RI), resistance impedance (S/D) and diameters of uterine (UA) and vaginal (VA) arteries. Steroid hormones were also assayed. Transrectal ultrasonography (TUS) was performed at 32 and 60 days to confirm the pregnancy per artificial insemination (P/AI).

**Results:**

The uterine PSV, TAMV, and PV were greater at the time of the cloprostenol sodium and second GnRH injections (*p<*0.05) than at the time of the first GnRH injection. The vaginal PSV, PV were greater at the time of the cloprostenol sodium than at the time of the first and second GnRH injections (*p<*0.05). The receiver operating characteristic curve (ROC curve) indicated a high correlation between the uterine and vaginal blood flow and the rate of the pregnancy (*p<*0.05). The area under the ROC curve was 0.920 and 0.87 (*p<*0.05) for vaginal and uterine arteries respectively at time of G2. The serum levels of progesterone, estrogen and cortisol were correlated with the P/AI (*p<*0.05). The P/AI significantly decreased from 43.9 % at 32 d to 35.37 % at 60 d.

**Conclusion:**

These results indicate that noninvasive Doppler ultrasonography is a valid method to evaluate changes in the characteristics of uterine and vaginal blood flow in cows during the Ovsynch protocol. Furthermore, vaginal and uterine blood flow are two determinant factors for the higher conception rates in Holstein dairy cows.

## Background

The profitability of dairy cow operations is dependent mainly on reproductive performance, and reproductive management is one of the most important factors for increasing the profitability of dairy enterprises. Calving interval, milk production efficiency, herd replacement dynamics, and timing of the establishment of pregnancy during lactation impact the profitability of dairy herds [1]. Successful artificial insemination (AI) and conception rates after a voluntary waiting period (VWP) are two main determinants of time to the establishment of pregnancy postpartum. The VWP has traditionally been 60 days in dairy farms [2]. Dairy farm revenue is affected positively by maximizing the number of female calves born for replacements in the herd, minimizing replacement of cows due to reproductive disorders, and sustaining the length of the lactation curve when milk production is greatest [3].

Although there are numerous reproductive management strategies available for dairy farms, implementation of the best management program remains a major challenge for dairy producers owing to the complicated interactions of multiple biological and management factors affecting dairy herd dynamics and economics [4, 5]. The dairy industry has approved hormonal protocols for synchronizing estrus and ovulation as reproductive management systems to increase the overall reproductive performance of lactating cows [6]. Ovsynch protocols are important for increasing the use of AI with semen from bulls with desired genetic with respect to milk production daughters, but AI does not improve fertility in dairy cattle as conception rates (CR) for AI services after Ovsynch are similar to those for cows inseminated after observed natural estrus [4, 7]. New protocols, such as Presynch-Ovsynch and Double-Ovsynch, have been developed to improve the timing of insemination and fertility following AI at a timed AI (TAI) [8–10].

Doppler ultrasonography has broadened the utility of imaging from an anatomical to a physiological base [11]. This novel method has been used to assess healthy and pathological variations in uterine blood flow [12]. Earlier research, utilizing invasive techniques, evaluated blood flow in the reproductive system of cows to find rhythmic alterations related to changing concentrations of progesterone (P4) and estradiol (E2) in serum during the estrous cycle [13–15]. Color Doppler ultrasonography, a non-invasive technology developed two decades ago, is able to detect variations in blood flow throughout the estrous cycle of mares [16], sheep [17], and cows [18]. It has also been used to assess changes in blood flow during pregnancy [19, 20], uterine torsion [21]; and the puerperium [22, 23]. This imaging approach can be used to assess the vasculature of ovarian follicles [24], corpora lutea (CL) [25], ovulatory follicles [26], and the uterus [27].

To the best of our knowledge, uterine and vaginal blood flow in cows has not yet been studied during the estrus synchronization regime, and no attempts have been made to correlate the changes in the blood flow with subsequent pregnancy yield. The purpose of this study was to evaluate uterine and vaginal blood flow in cows undergoing an Ovsynch program for estrus synchronization and to investigate the relationship between the changes in uterine and vaginal blood flow and steroid hormone levels and pregnancy rates in Holstein dairy cows.

## Results

### Ovulation and pregnancy per insemination parameters

Pregnancy per AI at 32 and 60 d after AI and pregnancy loss are presented in Table 1. The overall P/AI was 43.9 % (36/82) and 35.37 (29/82) for the first and second pregnancy checks, respectively (*P* = 0.001). All experimental cows were ovulated within 33±4.2 h after the second injections of GnRH.

**Table 1:** Pregnancy rate per artificial insemination (P/AI) for Holstein dairy cows exposed to GPG ovsynch program

**Pregnancy check**	**parity**		
	**Prim-parous**	**Pleuri-parous**	**overall**
32±3	41.9 (13/31)	45.09 (23/51)	43.9 (36/82)
60±3	32.25 (10/31)	37.2(19/51)	35.37 (29/82)
*P* value	0.002	0.01	0.001

### The effects of GPG Ovsynch on uterine blood flow

**Fig. 1:**
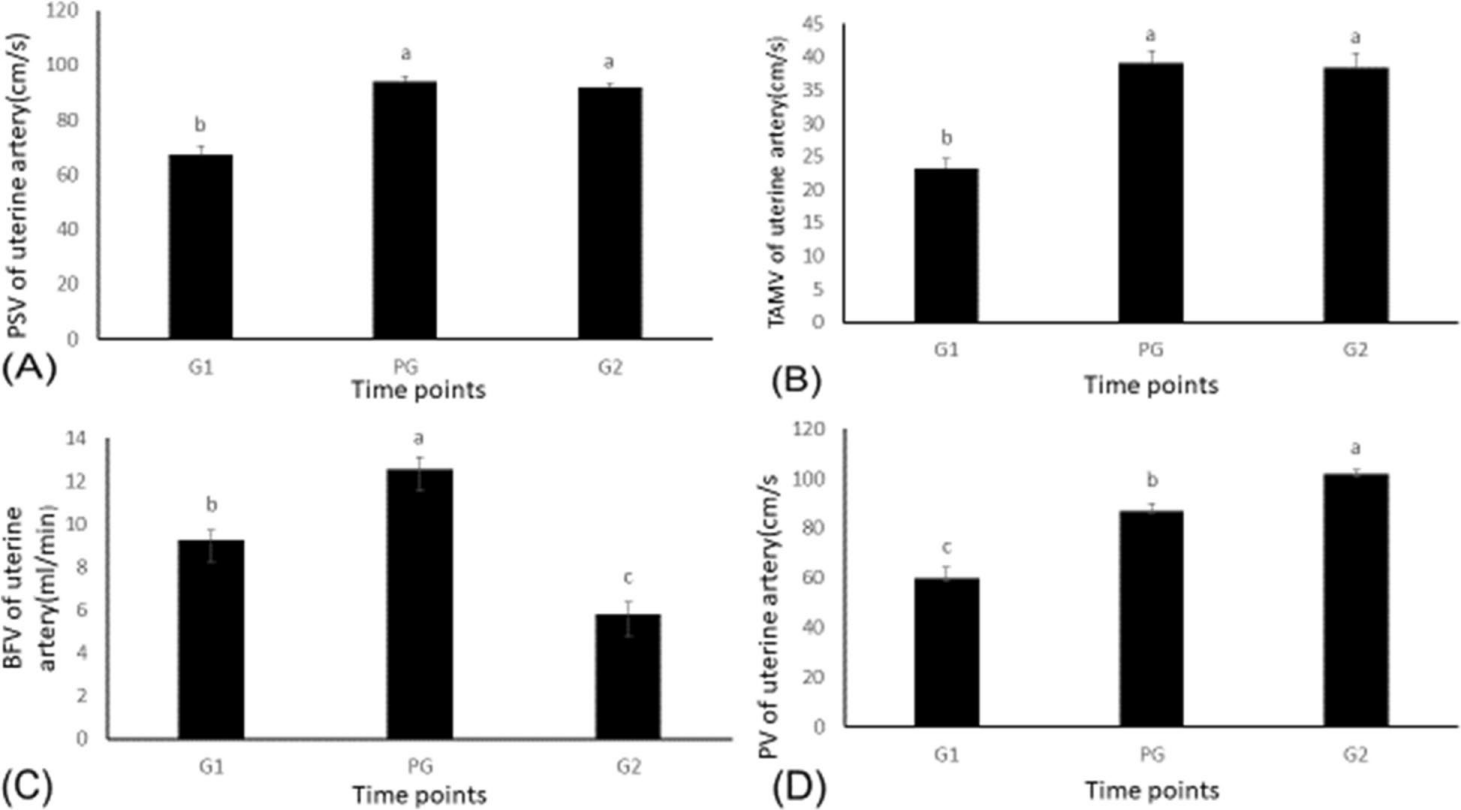
Peak systolic velocity (PSV) in the uterine artery, time-averaged maximum velocity (TAMV), blood flow volume (BFV), peak velocity (PV) changes in response to the GnRH:PGF2α management program. Values are means ± standard error of mean (SEM). Means with different superscripts (**a**, **b**, **c**) are significantly different (*P <* .05)

**Fig. 2:**
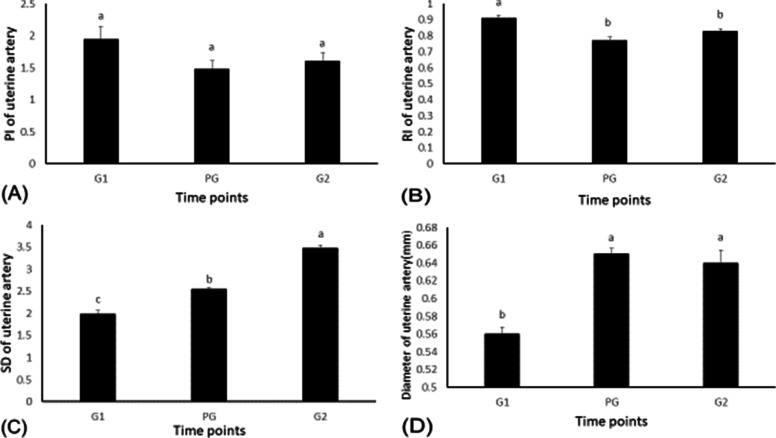
Changes in pulsatility index (PI), resistance index (RI), systolic/diastolic(S/D), and diameter (D) changes in the uterine artery in response to the GnRH:PGF2α management program. Values are means ± standard error of mean (SEM). Means with different superscripts (**a**, **b**, **c**) are significantly different (*P <* .05)

Imaging of the uterine blood vessel was successful in all of our examinations. Blood flow parameters PSV, TAMV, BFV, PV (Fig. 1 A, B, C, D respectively), RI, PI, S/D, and uterine artery diameter (Fig. 2 A, B, C, D respectively) during the GPG management program for estrus synchronization in Holstein dairy cows underwent interesting changes. Uterine blood flow PSV was increased (*P <* 0.0001) at the time during which cows were injected with PGF2α and then the second injection of buserelin (94.01 ± 2.02 and 92.03 ± 1.15 cm/s, respectively) when compared with values at the time of the first injection of buserelin (67.26 ± 3.09cm/s). Similarly, the TAMV of the uterine blood flow increased (*P <* 0.05). The maximum increase occurred with the PGF2α and the second injection of buserelin acetate (39.09 ± 1.86 vs. 38.35 ± 2.14 cm/s) as compared to the time of the first injection of buserelin (23.22 ± 1.52 cm/s). In the same way, the peak velocity (PV) was increased (*P <* 0.0001) at the time during which cows were injected with PGF2α, and then the second injection of buserelin (87.12 ± 2.93 and 101.66 ± 1.93 cm/s, respectively) when compared with values at the time of the first injection of buserelin (59.72 ± 4.61cm/s). The uterine artery blood flow volume (U_BFV) increased (*P <* 0.05) during the time when PGF2α was administered (12.58 ± 0.54 ml/min) compared to the times for the first and the second injections of buserelin (9.25 ± 0.47 and 5.83 ± 0.53 ml/min, respectively). The resistance impedance Doppler indices (RI, PI, S/D) for uterine blood flow during the GPG protocol for synchronization of estrus changed significantly. The resistance index (RI) and pulsatility index (PI) for uterine blood flow increased (*P <* 0.05) during the time of the first injection of buserelin (1.95 ± 0.02 vs. 0.91 ± 0.2, respectively) compared to the time of the PGFα injection (1.48 ± 0.02 vs. 0.77 ± 0.13, respectively) and the second injection of buserelin acetate (1.61 ± 0.01 vs. 0.83 ± 0.13, respectively). On the other hand, the S/D ratio of uterine blood flow increased (*P <* 0.05) at the time of the second injection of buserelin acetate (3.48 ± 0.07) compared to the time of the first injections of buserelin acetate and PGF2α (1.99 ± 0.08 and 2.54 ± 0.05, respectively). The diameter of the uterine artery (D) increased (*P <* 0.05) in response to PGFα and the second injection of buserelin acetate (0.65 ± 0.01 and 0.64 ± 0.01 mm, respectively) compared to the time of the first injections of buserelin acetate (0.56 ± 0.01mm).

As illustrated in Table 2, concentrations of progesterone in serum were positively correlated with volume of blood flow through the uterine artery and TAMV (BFV: *r*2 = 0.66, *P <* 0.01; TAMV: *r*2 =0.63, *P <* 0.05, respectively) and negatively correlated with the RI (RI: *r*2 = -0.50, *P <* 0.05). As for concentrations of cortisol in serum, they were positively correlated with U_BFV and TAMV (BFV: *r*2= 0.77, *P <* 0.01; TAMV: *r*2 =0.5, *P <* 0.05, respectively), but negatively correlated with RI (RI: *r*2=-0.51, *P <* 0.05) and not correlated with PI (P > 0.05). There was a negative correlation between concentrations of E2 in serum and U_BFV and TAMV (BFV: *r*2= -0.38, *P <* 0.15; TAMV: *r*2 = -0.7, *P <* 0.01, respectively), but no correlation with uterine artery PI (*P >* 0.05).

**Table 2:** Pearson’s rank correlation coefficients for the relationships between concentrations of estrogen, progesterone and cortisol in serum and peak systolic velocity (PSV), blood flow volume (BFV), time-averaged maximum velocity (TAMV), pulsatility index (PI), resistance index (RI), systolic/diastolic velocity (S/D), and peak velocity (PV) with respect to blood flow through uterine artery (U) based on parameters measured using Doppler ultrasonography (D Parameters) in Holstein cows during the GnRH- PGF2α management program. Bold parameters are *R* value and reverse bolding indicates *P* value

	**P4**	**E2**	**Cortisol**	**U-PSV**	**U-TAMV**	**U-BFV**	**U-PI**	**U-RI**	**U-SD**	**U-D**
**P4**	**1**	**-0.18**	0.94**	**-0.17**	0.63*	0.66**	**0.04**	**-0.5**	**-0.49**	**0.39**
P4		0.5	0.000	0.5	0.01	0.007	0.8	0.05	0.05	0.14
**E2**	**-0.18**	**1**	**-0.26**	**0.02**	**-0.7**	**-0.38**	**0.08**	**0.27**	**0.05**	**-0.20**
E2	0.5		0.34	0.93	0.1	0.15	0.7	0.31	0.85	0.4
**Cortisol**	0.94**	**-0.26**	**1**	**-0.23**	**0.5**	0.77**	**0.19**	-0.51*	**-0.12**	**0.38**
cortisol	0.000	0.34		0.4	0.05	0.001	0.47	0.05	0.65	0.15
**U-PSV**	**-0.17**	**0.02**	**-0.23**	**1**	**0.14**	-0.52*	**-0.21**	**-0.23**	**0.43**	**-0.07**
U-PSV	0.5	0.93	0.4		0.6	0.04	0.43	0.4	0.1	0.7
**U-TAMV**	0.63*	**-0.7**	0.5*	**0.14**	**1**	**0.9**	**-0.5**	**-0.37**	0.66**	**0.19**
U-TAMV	0.01	0.1	0.05	0.61		0.01	0.05	0.1	0.006	0.47
**U-BFV**	0.66**	**-0.38**	0.77**	-0.5*	**0.9**	**1**	**-0.49**	**-0.34**	-0.5*	**0.22**
U-BFV	0.007	0.15	0.001	0.04	0.01		0.05	0.2	0.61	0.41
**U-PI**	**0.04**	**0.08**	**0.19**	**-0.2**	**-0.5**	**-0.49**	**1**	**-0.21**	-0.85**	**0.2**
U-PI	0.8	0.75	0.4	0.43	0.05	0.05		0.66	0.00	0.46
**U-RI**	**-0.5**	**0.27**	-0.51*	**-0.23**	**-0.37**	**-0.34**	**0.12**	**1**	**-0.07**	**-0.19**
U-RI	0.05	0.31	0.05	0.4	0.16	0.2	0.66		0.78	0.48
**U-S/D**	**-0.49**	**0.05**	**-0.12**	**0.43**	0.66**	-0.56*	-0.85**	**-0.07**	**1**	**0.05**
U-S/D	0.05	0.85	0.65	0.1	0.006	0.02	0.00	0.7		0.85
**U-D**	**0.39**	**-0.20**	**0.38**	**-0.07**	**0.19**	**0.22**	**0.2**	**-0.16**	**-0.05**	**1**
U-D	0.14	0.47	0.15	0.7	0.4	0.4	0.46	0.4	0.8	

^*^P<0.05; **P<0.01

### The effects of GPG Ovsynch on vaginal blood flow

**Fig. 3:**
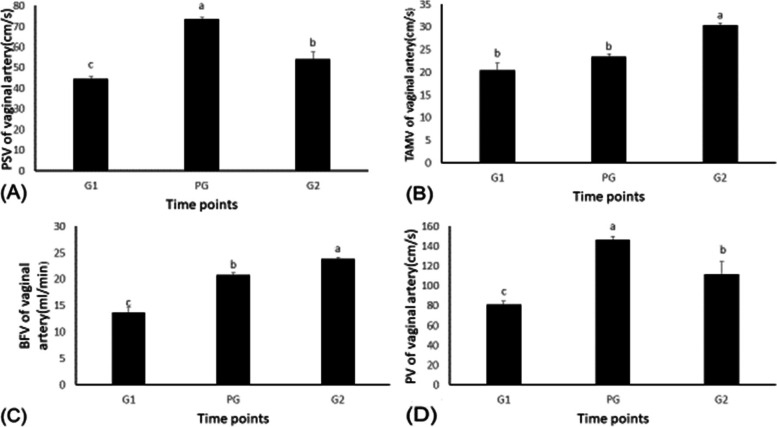
Changes in peak systolic velocity (PSV), time-averaged maximum velocity (TAMV), blood flow volume (BFV), and peak velocity (PV) in the vaginal artery in response to the GnRH:PGF2α management program. Values are means ± standard error of mean (SEM). Means with different superscripts (**a**, **b**, **c**) are significantly different (*P <* .05)

**Fig. 4:**
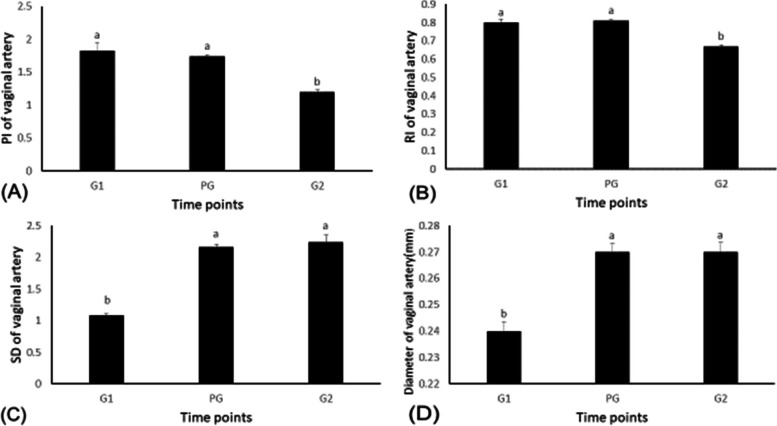
The pulsatility index (PI), resistance index (RI), systolic/diastolic(S/D), and changes in diameter (**D**) of the vaginal artery in response to the GnRH:PGF2α management program. Values are means ± standard error of mean (SEM). Means with different superscripts (**a**, **b**) are significantly different (*P <* .05)

The Doppler parameters and the diameter of the vaginal artery changed during GPG ovsynch protocol (Figs. 3 and 4). Vaginal blood flow peak systolic velocity (PSV) and peak velocity (PV) increased (*P <* 0.05) at the time of injection of PGF2α (73.51 ± 0.93 and 146.5 ± 3.41 cm/s) compared to times of the first and the second injections of buserelin acetate (44.63 ± 1.05 vs 81.22 ± 3.42 and 54.28 ± 3.38 vs 111.45 ± 12.9 cm/s, respectively), as was TAMV of vaginal blood flow (*P <* 0.05). The maximum increase occurred at the second injection of buserelin acetate (30.39±0.42 cm/s) when compared to times of the first injection of buserelin and the injection of PGF2α (20.45 ± 1.59 and 23.22 ± 0.62 cm/s, respectively). Vaginal blood flow during the GPG estrus synchronization protocol changed significantly. The resistance index (RI) and the pulsatility index (PI) of the vaginal blood flow increased (*P <* 0.05) during the time of the first injection of buserelin (0.8 ± 0.01 vs. 1.82 ± 0.13, respectively) and the time when PGF2α was injected (0.81 ± 0.04 vs. 1.74 ± 0.02, respectively) compared to time of the second injection of buserelin acetate (0.67 ± 0.009 vs. 1.2 ± 0.03, respectively). Moreover, the S/D ratio of vaginal blood flow increased (*P <* 0.05) in response to PGF2α and the second injection of buserelin acetate (2.16 ± 0.04 and 2.24 ± 0.1, respectively) as compared to time of the first injection of buserelin acetate (1.08 ± 0.02). Additionally, the diameter of the vaginal artery (D) increased (*P <* 0.05) in response to the injection of PGF2α and a second injection of buserelin acetate (0.27 ± 0.003 and 0.27 ± 0.004 mm, respectively) compared to the first injection of buserelin acetate (0.24 ± 0.004 mm). As showed in Table 3, There was a positive correlation between the vaginal blood flow peak systolic velocity (PSV) and concentrations of both progesterone (PSV: *r*2= 0.75, *P <* 0.01) and cortisol in serum (PSV: *r*2= 0.76, *P <* 0.01). In contrast the concentrations of both progesterone and cortisol in serum were negatively correlated with vaginal blood flow resistance index (RI) (RI: *r*2= -0.44, *P >* 0.05 and RI: *r*2= -0.37, *P <* 0.1, respectively) and also with PI (PI: *r*2= -0.48, *P >* 0.05 and PI: *r*2= -0.54, *P <* 0.1, respectively). Concentrations for E2 in serum were negatively correlated with the vaginal artery TAMV and BFV (TAMV: *r*2= - 0.09; BFV: *r*2= -0.17, *P <* 0.5, respectively).

**Table 3:** Pearson's rank correlation coefficients for the relationships between concentrations of estrogen, progesterone and cortisol in serum and peak systolic velocity (PSV), blood flow volume (BFV), time-averaged maximum velocity (TAMV), pulsatility index (PI), resistance index (RI) systolic/diastolic velocity (S/D), and peak velocity (PV) of blood flow through vaginal artery (V) using Doppler ultrasonography parameters (D) in Holstein cows during the GnRH PGF2α management program. Bold parameters are *R* value and reverse bolding indicates *P* value

	P4	E2	Cortisol	V-PSV	V-TAMV	V-BFV	V-PI	V-RI	V-SD	V-D
**P4**	**1**	**-0.18**	0.94**	0.75**	0.98**	**0.33**	**-0.48**	**-0.44**	0.69**	**0.49**
**P4**		0.5	0.000	0.001	0.01	0.22	0.06	0.09	0.01	0.06
**E2**	**-0.18**	**1**	**-0.26**	**-0.31**	**-0.09**	**-0.17**	**-0.1**	**0.18**	**-0.04**	**-0.27**
**E2**	0.5		0.34	0.25	0.7	0.047	0.7	0.5	0.86	0.32
**Cortisol**	0.94**	**-0.26**	**1**	0.76**	**-0.05**	**0.26**	-0.54*	**-0.37**	**0.12**	**0.37**
**cortisol**	0.000	0.34		0.001	0.83	0.34	0.03	0.16	0.64	0.17
**V-PSV**	0.75**	**-0.31**	0.76**	**1**	**-0.13**	**0.1**	**0.34**	**-0.31**	**0.4**	**0.41**
**V-PSV**	0.001	0.25	0.001		0.62	0.7	0.2	0.24	0.1	0.12
**V-TAMV**	**0.98**	**-0.17**	**-0.05**	**-0.13**	**1**	0.74**	-0.81*	**-0.34**	- 0.74*	**0.5**
**V-TAMV**	0.01	0.7	0.83	0.62		0.001	0.01	0.21	0.01	0.05
**V-BFV**	**0.33**	**-0.17**	**0.26**	**0.1**	0.74**	**1**	**0.02**	**-0.3**	**0.25**	0.82**
**V-BFV**	0.22	0.047	0.34	0.7	0.001		0.9	0.26	0.35	0.000
**V-PI**	**-0.48**	**-0.1**	-0.54*	**0.34**	-0.81*	**0.02**	**1**	**-0.21**	**0.4**	**-0.04**
**V-PI**	0.06	0.72	0.03	0.2	0.01	0.9		0.43	0.13	0.88
**V-RI**	**-0.44**	**0.18**	**-0.37**	**-0.31**	**-0.34**	**-0.3**	**-0.21**	**1**	**0.18**	**-0.46**
**V-RI**	0.09	0.5	0.16	0.24	0.74*	0.26	0.43		0.5	0.08
**V-S/D**	0.69**	**-0.04**	**0.12**	**0.4**	**0.01**	**0.25**	**0.4**	**0.18**	**1**	**0.26**
**V-S/D**	0.01	0.8	0.64	0.1	0.52	0.35	0.13	0.5		0.33
**V-D**	**0.49**	**-0.27**	**0.37**	**0.41**	**0.5**	0.82**	**-0.04**	**-0.46**	**0.26**	**1**
**V-D**	0.06	0.32	0.17	0.12	0.05	0.000	0.88	0.08	0.33	

^*^P<0.05; **P<0.01

### The effects of the GPG Ovsynch protocol on concentrations of cortisol, progesterone, and estrogen in serum

As summarized in Table 4, concentrations of P4 in serum increased during the luteal growth phase of the Ovsynch protocol at the time when cloprostenol sodium was injected (10.43 ± 0.17 ng/dl, *P <* 0.05) as compared to times of the first and the second injections of buserelin acetate (0.54 ± 0.17 and 2.07 ± 1.12 ng/dl, respectively). In contrast, concentrations of E2 in serum increased (*P <* 0.05) during the time of the first injection of buserelin acetate (21.41 ± 0.99 pg/dl) and increased further during the time of the second injection of buserelin acetate (25 ± 1.83 pg/dl) compared to the time of the PGF2α injection (16.56 ± 0.78 pg/dl). Furthermore, concentrations of cortisol in serum increased (*P <* 0.05) at the time of the PGF injection (1.08 ± 0.02 ng/dl) when compared to the times of the first and second injections of buserelin acetate (0.49 ± 0.04 and 0.5 ± 0.02 ng/dl, respectively).

**Table 4:** Concentrations (means + standard error of mean (SEM)) of estrogen, progesterone and cortisol in serum from Holstein cows at the time of the initial treatments with GnRH and PGF2α in the GPG program

**Time points**	**Hormonal levels**		
	**Progesterone (ng/dl)**	**Estrogen (pg/dl)**	**Cortisol (ng/dl)**
**First GnRH injection**	0.54 ±0.17^b^	21.41 ±0.99^a^	0.49 ±0.04^b^
**PGF2α injection**	10.43 ± 0.17^a^	16.56 ± 0.78^b^	1.08 ± 0.02^a^
**Second GnRH injection**	2.07 ± 1.12^b^	25 ± 1.83^a^	0.5 ±0.02^b^

Concentrations (means ± standard error of mean (SEM)) of estrogen, progesterone and cortisol in serum from Holstein cows at the time of the initial treatments with GnRH and PGF2α in the GPG program. Means with different superscripts (a,b) are significantly different between time points (*P* < 0.05)

### Prediction of pregnancy

**Fig. 5:**
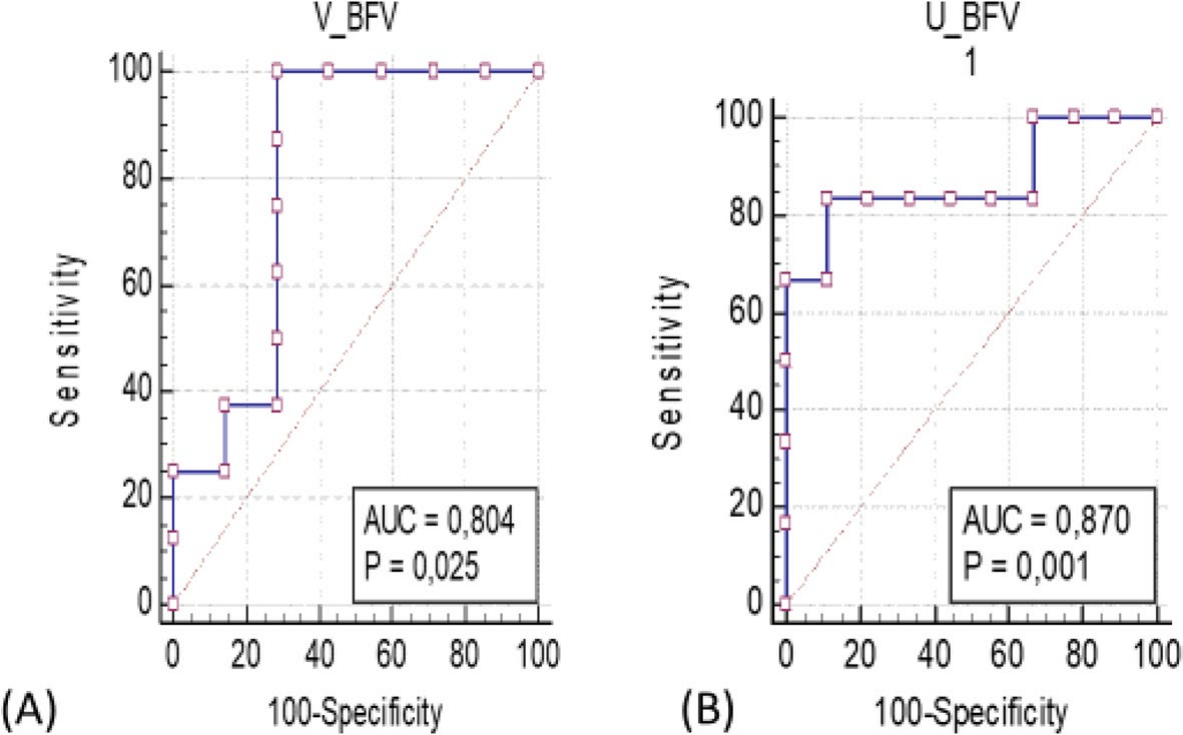
ROC curve for predicting viable pregnancy by (**A**) uterine artery blood flow volume (U_BFV), (**B**) vaginal artery blood flow volume (V_BFV) at the time of g2 shots. The ROC curve was constructed by plotting the true positive rate (sensitivity) on the y-axis and the false positive rate (1-specificity) on the x-axis

**Fig. 6:**
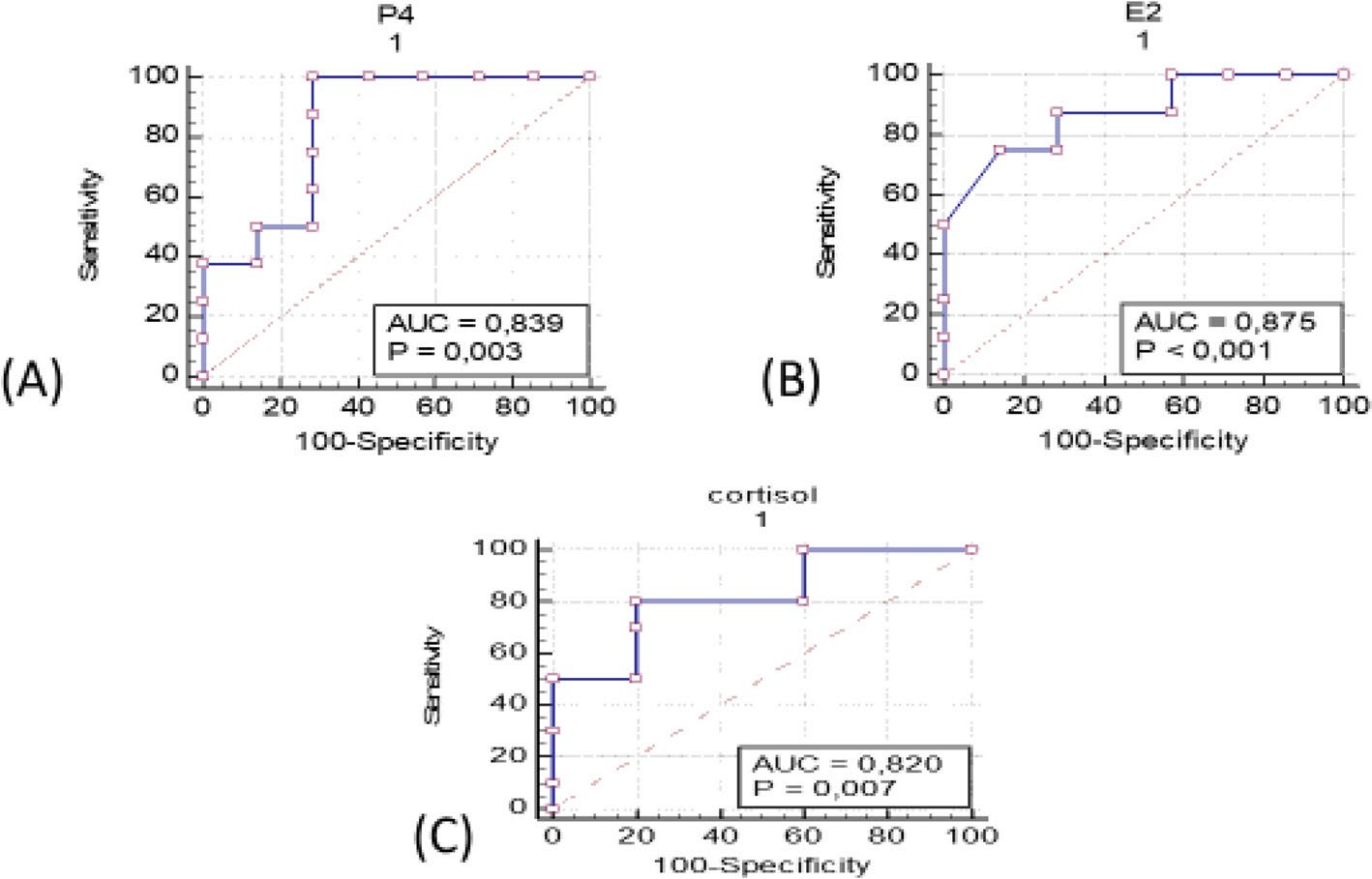
ROC curve for predicting viable pregnancy by (**A**) serum progesterone P4, (**B**) serum estrogen E2 and (**C**) serum cortisol concentrations at the time of G2 shots. The ROC curve was constructed by plotting the true positive rate (sensitivity) on the y-axis and the false positive rate (1-specificity) on the x-axis

ROC curves were constructed and the area under the curves could be seen in (Figs. 5 and 6). It was possible to set a cutoff value for every parameter taken into account (Table 5). The area under the curve examining the P4 level at the time of G2 shot as a predictor of pregnancy was found to be 0.839 and the best P4 cutoff value was 0.71 ng/ml with (sensitivity of 90% and a specificity of 37.5%; *P <* 0.003). The volume of vaginal artery blood flow volume (V_BFV)as a good predictor of pregnancy. At the time of the G2 shot, the V_BFV cutoff value was set at 0.90 ml/min with an increase in sensitivity (90.8%) and specificity (30.8%; *P <* 0.001). Regarding the other parameters, it was possible to set a cutoff value for U_BFV, cortisol, at the time of G2 shot that was (0.722 ml/min and 0.60 ng/ml, respectively), with a (sensitivity of 90% and 80% and a specificity of (33.33% and 40% ;respectively) (*P <* 0.01, 0.007 ;respectively). The area under the curve (AUC) examining the E2 level at the time of G2 shot as a predictor of pregnancy was found to be 0.875 and the best E2 cutoff value was 0.607 Pg/ml with (sensitivity of 87.5% and specificity of 42.86%; *P <* 0.001). ALso, AUC for uterine and vaginal blood flow were 0.870 and 0.804 respectively at the time of injection of G2.

**Table 5:** Receiver operating characteristic (ROC) curve of uterine blood flow, vaginal blood flow, serum progesterone, serum estrogen and serum cortisol at the G2 shots

**Items**	**Cutoff value**	**AUC value**	**Sensitivity (%)**	**Specificity (%)**	***P*-value**
U_BFV	0.722 ml/min	0.870 ± 0.115	90%	33.33%	*P <* 0.001
V_BFV	0.90 ml/min	0.804 ± 0.07	90.8%	30.8%	*P <* 0.03
Cortisol	0.60 ng/ml	0.820 ± 0.119	80%	40%	*P <* 0.007
Estrogen	0.607 pg/ml	0.875 ± 0.09	87.5%	42.86%	*P <* 0.001
Progesterone	0.7143 ng/ml	0.839 ± 0.116	90%	37.5%	*P <* 0.003

## Discussion

Transrectal color Doppler ultrasonography is a noninvasive method used to investigate the effects of a gonadotropin treatment during superovulation on uterine blood flow, as well as its relationship with steroid hormone levels, ovarian response, and the yield of embryos in dairy cows [28]. The present study characterized, for the first time, changes in uterine and vaginal blood flow during the period of application of the Ovsynch protocol in Holstein dairy cows.The P/AI was correlated significantly with the vaginal blood flow at time of insemination than the uterine blood flow according to the ROC curves analyses. The present study has high economic value because it guides the veterinarian to either give the injections of GnRH and/or PGF2α during the Ovsynch protocol on dairy farms. The results of the current study revealed significant variations in uterine and vaginal blood flow indices in Holstein cows undergoing the GPG estrus synchronization regime as well as P/AI. The overall P/AI in the current study was 43.09% and 35.37% at the first and second pregnancy checks. The decreased pregnancy percentage may be attributed to the decreased uterine and vaginal blood flow at the time of artificial insemination. This hypothesis was proved by the results of ROC curve analyses that indicated a positive correlation between the blood flow of both uterine and vaginal arteries and the success of the pregnancy at day 32 after TAI.

The effects of the first and second injections of buserelin acetate, as well as PGF2α significantly affected uterine blood flow. The peak systolic velocity in uterine blood flow and the TAMV of uterine blood flow were measured during the time of the GPG treatments. When comparing the first and second injections of buserelin, uterine artery BFV increased during the time of the PGF2α injection, indicating that blood flow increased at the time of AI and during the luteal phases of the estrous cycle [29]. Also, uterine blood flow increases during the proestrus and estrus phases of the estrous cycle of dairy cows [18]. Blood flow velocity is relatively constant during diestrus in response to high concentrations of progesterone [11]. Similarly, uterine blood flow in the present study was significantly positively correlated with concentrations of E2 and P4 in serum.

In the present study, concentrations of cortisol in serum were greater at the time of PGF2α injections than during the first and second injections of buserelin. The pattern of changes in the serum cortisol was similar to those reported previously for cows and mares. Prolactin and cortisol were present in greater concentrations in the blood during estrus in cows, while concentrations of cortisol were greater during the luteal phase of the estrous cycle in mares [30, 31].

In the current study, the resistance impedance indices (RI, PI, S/D) for uterine blood flow changed significantly in response to the G:PG program. When compared to the effects of injection of PGF2α and the second injection of buserelin acetate, the RI and PI of uterine blood flow increased significantly in response to the first injection of buserelin. The S/D ratio of uterine blood flow increased at the time of the second injection of buserelin. The variation in resistance impedance indices in the uterine arteries may be attributed to changes in concentrations of P4, E2 and cortisol in serum as well as changes in the diameter of the uterine arteries [18, 28]. Previous research established that increases in estradiol in plasma act as the primary vasodilator of the uterine artery, resulting in an increase in uterine blood flow [28, 32]. Similarly, changes in indices of uterine artery blood flow (PSV, PV, TAMV) were positively correlated with BFV ([11, 20].

It is worth noting that previous research has shown that RI increases during proestrus [18, 33]. During early estrus, the resistance index was strongly correlated with both the average maximum velocity and volume of blood flow in the uterine artery to perfuse the uterus. The highest correlation was found on days 0 and 1 of the estrous cycle, which corresponded to the first and second injection of buserelin in the current study [34]. On the other hand, they found a negative relationship between RI of uterine blood flow and concentrations of estradiol in serum, but the correlation between RI and concentrations of progesterone in serum was not significant. In the current study, there was a negative correlation between RI in the uterine artery and serum progesterone levels, but there was not a significant correlation with serum estradiol levels. Furthermore, concentrations of cortisol in serum were correlated positively with volume and TAMV for uterine arterial blood flow, negatively correlated with RI and S/D, and not significantly correlated with PI. These findings suggest that uterine blood flow changes at different times during the GPG program in dairy cows when there are changes in concentrations of steroid hormones in serum.

In this study, changes in vaginal artery blood flow in dairy cows were investigated for the first time during the G:PG Ovsynch program. A previous study of pregnant buffalo characterized changes in vaginal blood flow during pregnancy [35].

In the current study, changes in vaginal blood flow during the G:PG Ovsynch estrus synchronization program were significant. The PSV and PV for vaginal blood flow increased more at the time of PGF2α injections than at the time of the first and second injections of buserelin acetate. The increase in blood perfusion via the vaginal artery was characterized by decreasing PI and RI values in response to effects of gonadotropins on the ovaries (follicle development) and/or the increase in concentrations of estradiol in serum [28]. During estrus, estradiol acts as a major vasodilator, and there is an increase in blood flow as its concentrations increase in serum [28, 32].

When compared to the second injection of the buserelin acetate, the RI and PI for vaginal blood flow were significantly greater than during the first injection of buserelin and the injection of PGF2α. Furthermore, the S/D ratio of vaginal blood flow was greater in response to PGF2α and the second injection of buserelin acetate as compared to the response to the first injection of buserelin acetate. It is likely that the increase in vaginal blood flow in cows following gonadotropin treatment is due to the vasodilatory effect of increasing concentrations of estradiol, despite the fact that the increase in estradiol in serum was greater than the increase in BFV and decrease in PI, respectively [28, 36].

This is the first study in dairy cows to demonstrate the relationship between vaginal blood flow and concentrations of steroid hormones in serum during treatment with gonadotropins and PGF2α during the Ovsynch protocol. The concentrations of progesterone and cortisol in serum were correlated positively with PSV and BFV, but negatively correlated with RI and PI. These consistent changes in concentrations of steroids in serum likely account for the fluctuations in vaginal blood flow in Holstein dairy cows during the G:PG program. These valuable results may be of economic importance at different times of the Ovsynch protocol with respect to either deciding to continue with the cows until they are inseminated or reinitiating the protocol without inseminating the cows. Accordingly, this may improve the reproductive performance of Holstein dairy cows.

Regarding the relationship between the uterine and vaginal arteries blood flow changes, the current study is considered as the first one indicating that the vaginal blood flow is correlated with the uterine blood flow changes and this may explain the difference in the conception and pregnancy rates at time of insemination. These changes are controlled in general by the concentrations of steroid hormones during different times of gonadotropin and/or prostaglandin injections during the ovsynch program in dairy cows.

## Conclusion

The results of the current study revealed that noninvasive color Doppler ultrasound is a cost-effective tool for monitoring responses of Holstein dairy cows to hormones used in the Ovsynch protocol that may influence fertility.

## Materials and methods

### Cows and farm management

The Animal Care and Use Committee of the Faculty of Veterinary Medicine, Mansoura University, approved all procedures performed on the cows (M/158).

Lactating Holstein cows (*n* = 82) from the Dairy Unit of the Nobaria (Beheira Governorate) were enrolled between November 2021 and February 2022. Cows were housed in naturally ventilated barns with 10 rows of free stalls. Deep-bedded sand stalls, cooling fans over the feeding lane and, and sprinklers above the feed bunk for heat reduction during summer. The meal was designed to fulfil or surpass the nutritional needs of nursing Holstein cows that produce 45 kg of milk per day. All of the cows were provided with water ad libitum. All cows were managed strictly to minimize and control internal and external parasites, and they were dewormed on a regular basis. The cows were subjected to reproductive tests on a regular basis. The local veterinary authorities provided an annual vaccination system for the animals against endemic diseases such as foot and mouth disease, rift valley fever, and Pasteurella and clostridia. Cows were milked three times a day, at eight-hour intervals. The cows enrolled in the experimental procedures were about 60 ± 3 DIM.

### Study design

The Ovsynch procedure was used to synchronize lactating nonpregnant primiparous (*n* = 31) and multiparous (*n* = 51) Holstein cows at various DIMs. Estrus was synchronized using ovsynch protocol [4]. The experimental cows received 10 μg of a GnRH analogue (Buserelin acetate: Receptal®;MSD animal health, Egypt) intramuscularly followed 7 days later with an intramuscular injection of (500 μg cloprostenol sodium; Estrumate intramuscularly; Essex, Munich, Germany), and given 10 μg, injection of the GnRH analogue i.m. (2.5ml/animal) 48h after the PGF2aα treatment and the cows were bred 14-16 h then after. The ovaries were examined ultasonographically 12, 24, and 36 h later to confirm that ovulation has occurred. Day 1 of the estrous cycle was defined as the day when the dominant follicle was no longer detectable due to it having ovulated and the pregnancy check was done by ultrasound imaging at day 33 post inseminations.

### Blood samples and hormonal assays

After each ultrasonographic examination, blood samples from the coccygeal vein were taken. Within 1 h, serum (vacutainer tubes Plain®, Lab Supply, Egypt) was separated and frozen at -20 °C until analyzed. An established enzyme immunoassay was used to measure concentration of p4 in serum progesterone [37]. Concentrations of P4 in serum were measured in duplicate using a commercial solid-phase, no-extraction RIA (Coat-a-count, Diagnostic Products Corp., Los Angeles, CA; ImmuChem Coated Tube, MP Biomedicals, Costa Mesa, CA). To assess the assay's precision, control samples with high (6.0 ng/mL for) and low (0.3 ng/mL for) concentrations of P4 were analyzed. The sensitivity of the P4 assay was 0.03 ng/mL on average. The intra-assay coefficient of variation for samples with a high concentration of P4 was 10.6%, and 7.9% for low-concentration serum samples. The coefficient of variation for the samples was 4.9 percent.

### Assay for estradiol (E2) in serum

Benzene:toluene was used to extract E2 from serum and determine circulating concentrations of E2 concentrations. Duplicate samples were analyzed using a double antibody RIA. The analysis was carried out using a commercially available kit (MaiaZen Estradiol R-FA-120, Zen Tech SA, Liege, Belgium), as described previously [38]. The sensitivity of the assay was 0.3 pg/mL. A quality control sample (6.5 pg/mL E2) was included in quadruplicate. The intra-assay coefficient of variation was 16%.

### Concentrations of Cortisol in serum

Concentrations of cortisol in serum were determined using an enzyme immunoassay described previously [39]. The intra-assay and inter-assay coefficients of variation in high (*n* = 6) and low cortisol pooled serum samples (*n* = 5) were 6.0 percent and 11.4 percent, and 4.2 percent and 8.4 percent, respectively.

### Reproductive management and examinations of the reproductive tract using color Doppler ultrasonography

**Fig. 7:**
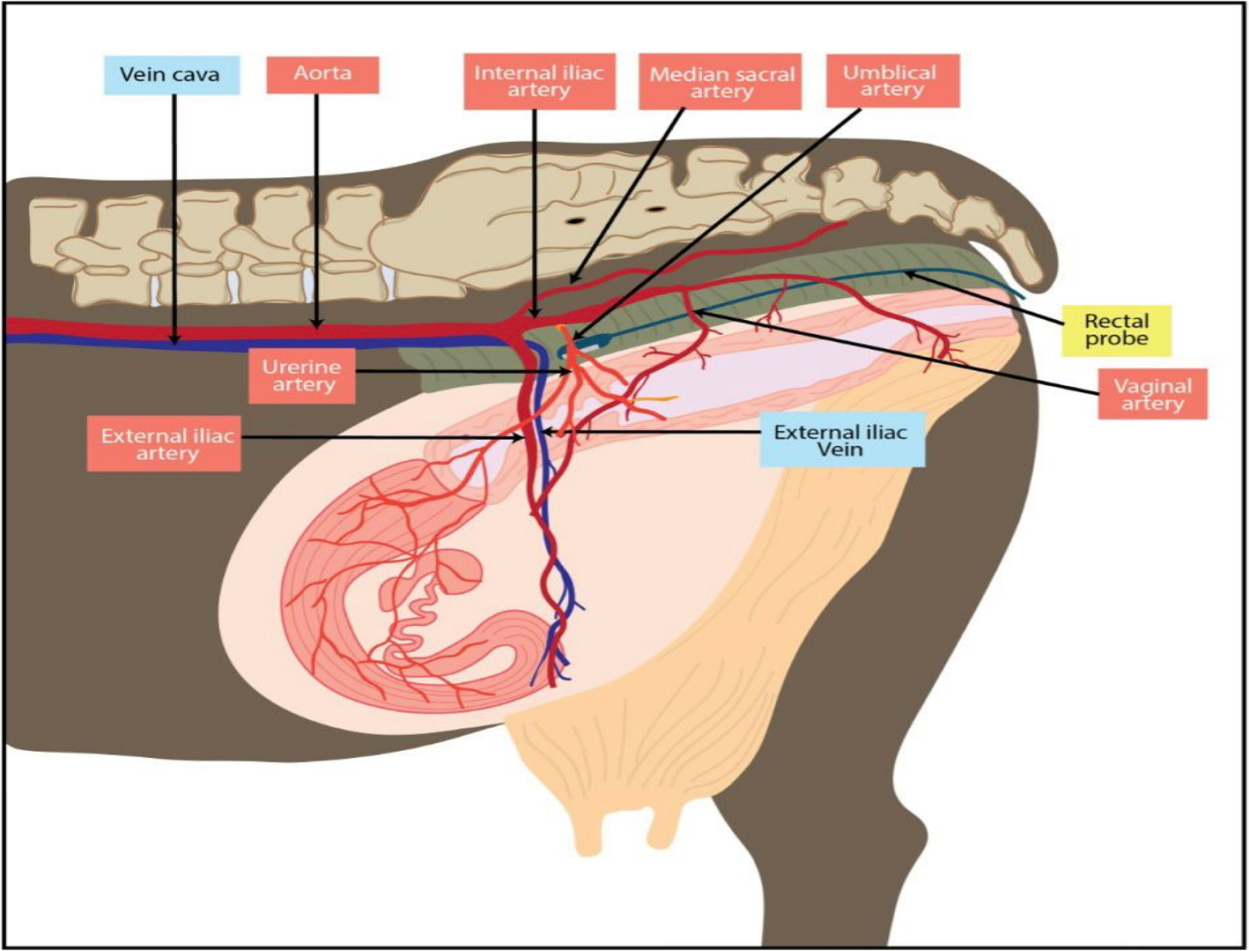
The uterine artery and vaginal artery are depicted diagrammatically to demonstrate the position for transrectal placement of an ultrasonography probe

Cows were enrolled at the time of the first GnRH injection in the Ovsynch protocol and were healthy during clinical and gynecological examinations. All management procedures were carried out while the cows were restrained in the feed bunk by self-locking head gates. The uterine arteries of Holstein dairy cows were located and examined in accordance with a previously established research protocol [11]. The rudimentary umbilical artery, located cranial to the external iliac artery, was used to examine the uterine artery, a branch originating from the internal iliac artery (Fig. 7). The Doppler waveforms were obtained at this location by activating the pulsed Doppler function and modifying the Doppler gate over the uterine artery to fit the vessel's diameter.

Except for the examination at the injection of second GnRH, performed between 18:00 and 20:00 p.m., all transrectal B-mode and color Doppler ultrasound examinations were performed by the same person (Heba Sharawy) between 7:00 and 11:00 a.m. All examinations were performed using the same ultrasound machine (Esaote MyLab 30X Vision, Esaote, Genova, Italy) with high-frequency linear transducers: 6–12 MHz with a filter of 100 Hz, power of 50%, pulse repetition frequency (PRF) of 4,500 Hz, and Doppler angle ranging from 0 to 40. To avoid continuous straining by the cows, an epidural anesthesia of 4 ml procaine hydrochloride (2% Procasel ®; Selectavet, Weyarn-Holzolling, Germany) was administered immediately before measuring blood flow.

The time-averaged maximum velocity (TAMV), resistance index (RI), pulsatility index (PI), resistance impedance (S/D), peak velocity (PV) and blood flow volume (BFV), as well as the diameters of the uterine and vaginal arteries, were the Doppler indices that the device displayed for each waveform when using the automatic mode as previously reported [35]. B-mode images were used to measure the diameters of the uterine and vaginal arteries.

### Pregnancy diagnosis

Transrectal ultrasound (TUS) was used to check for pregnancy in experimental cows 32±3 days after the first service if the cow hadn't been re-inseminated at detected estrus before. A positive pregnancy outcome was based on the amount of fluid in the uterus that was not echogenic and the size of the embryo compared to the expected stage of pregnancy. The fact that the embryo had a heartbeat was also used as proof that it was alive. The evidence of positive pregnancy was done by TUS 60±3 d after AI in all cows that were already known to be pregnant at the first examination unless the cow was left the herd.

### Statistical analyses

The data were presented as means ± SEM for statistical analysis using SAS® (version 9.2, SAS Institute). The Shapiro–Wilk test was used to determine the normality of all variables' distributions. To determine the effect of injection time on Doppler indices in uterine and vaginal arteries, as well as steroid hormone concentrations, a mixed model one-way analysis of variance was used, with time points as repeated measurements. Multiple pairwise comparisons were performed post hoc using Duncan's error rate adjustment.

An ROC-curve is constructed using the results was used to determine the cutoff point to determine the relationship between the uterine and vaginal blood flow and the pregnancy per artificial insemination (P/AI). The experimental cows were assigned as pregnant (positive outcome) or not pregnant (negative outcome) at day 60 post insemination for the purpose of running the ROC curve analyses. The ROC analysis option of MedCalc (version 12.5.0.0; MedCalc Software BVBA) was used to create the ROC curves. Differences were considered significant at *p* ≤ .05.

## Data Availability

The datasets used and analysed during the current study are available from the corresponding author on reasonable request.

## Abbreviations

AI: Artificial insemination
BVF: Blood flow volume
Cl: Corpus luteum
CR: Conception rate
D: Diameter of the artery
DIM: Days in milk
G1: Time of first gonadotropins injection
G2: Time of second gonadotropins injection
GnRH: Gonadotropin releasing hormone
E2: Estradiol
P4: Progesterone
(P/AI): Pregnancy per artificial insemination
PGF2α: Prostaglandin F2 alpha
PI: Pulsatility index
PSV: Peak systolic velocity
PV: Peak velocity
RI: Resistance index
ROC: Receiver operating curve
S/D: Systolic / Diastolic velocity
SEM: Standard error of mean
TAI: Time-artificial insemination
TAMV: Time-averaged maximum velocity
TUS: Transrectal ultrasonography
UA: Uterine artery
VA: Vaginal artery
VWP: Voluntary waiting period
